# Atezolizumab-Associated Autoimmune Diabetes Presenting as Diabetic Ketoacidosis in a Woman With Hepatocellular Carcinoma: A Case Report

**DOI:** 10.7759/cureus.108914

**Published:** 2026-05-15

**Authors:** Itizaz Hayat, Sajid Hayat, Taimoor Tahir, May Loo

**Affiliations:** 1 Internal Medicine, Queen Elizabeth Hospital, Gateshead, GBR; 2 Pulmonary Medicine, University Hospital Kerry, Tralee, IRL; 3 Medicine, Kuwait Teaching Hospital, Peshawar, PAK; 4 Surgery, Pakistan Institute of Medical Sciences, Islamabad, PAK; 5 Endocrinology and Diabetes, Queen Elizabeth Hospital, Gateshead, GBR

**Keywords:** atezolizumab, autoimmune diabetes, diabetic ketoacidosis (dka), hepatocellular carcinoma, immune-checkpoint-inhibitor, pd-l1 inhibitor, type 1 diabetes mellitus (t1dm)

## Abstract

Immune checkpoint inhibitor-related diabetes mellitus is a rare but clinically important endocrine adverse event that characteristically presents with abrupt-onset diabetic ketoacidosis, disproportionately normal or near-normal glycated haemoglobin, and evidence of autoimmune beta-cell destruction. We report the case of a 58-year-old woman receiving atezolizumab and bevacizumab for hepatocellular carcinoma who developed new-onset polyuria and polydipsia followed by diabetic ketoacidosis within weeks of commencing immunotherapy. Biochemical evaluation confirmed endogenous hyperinsulinaemia-unrelated hyperglycaemia with strongly positive islet autoantibodies, including glutamic acid decarboxylase (158 IU/mL) and zinc transporter 8 (1,467 U/mL), alongside a urinary C-peptide/creatinine ratio of 0.59 nmol/mmol, consistent with early but rapidly evolving beta-cell failure. She was treated with standard diabetic ketoacidosis care, transitioned to a basal-bolus insulin regimen with continuous glucose monitoring, and discharged with coordinated oncology-endocrinology follow-up. Atezolizumab was held during acute decompensation with plans for re-initiation once metabolic stability was achieved. This case adds to the growing literature on programmed death-ligand 1 inhibitor-induced diabetes in the hepatocellular carcinoma population, where such events are incompletely characterised. Clinicians managing patients on atezolizumab-based regimens should maintain a low threshold for glucose monitoring and early ketone testing in the presence of osmotic symptoms.

## Introduction

Immune checkpoint inhibitors (ICIs) have transformed the management of advanced malignancies by restoring immune surveillance against tumour cells. Atezolizumab, a humanised monoclonal IgG1 antibody targeting programmed death-ligand 1 (PD-L1), has become a standard of care for several solid malignancies. Since the landmark IMbrave150 trial, the combination of atezolizumab and bevacizumab has been the preferred first-line systemic treatment for unresectable hepatocellular carcinoma (HCC), demonstrating superior overall and progression-free survival over sorafenib [1].

The mechanism of action of atezolizumab - blockade of the PD-1/PD-L1 immune checkpoint - enhances cytotoxic T-cell activity against tumour cells but simultaneously disrupts peripheral immune tolerance, creating conditions for off-target autoimmunity [2]. Endocrine immune-related adverse events (irAEs) are among the more clinically significant complications of ICI therapy. Thyroid dysfunction, adrenal insufficiency, and hypophysitis are most commonly reported, but ICI-induced diabetes mellitus, though less frequent with an estimated incidence of approximately 0.2% across checkpoint inhibitor classes, is particularly dangerous owing to its rapid and often fulminant presentation [3]. Diabetic ketoacidosis (DKA) is a life-threatening metabolic emergency characterised by hyperglycaemia, ketonaemia, and metabolic acidosis arising from absolute or near-absolute insulin deficiency, and represents the most common acute manifestation of this complication.

In contrast to classic type 1 diabetes mellitus, ICI-induced diabetes tends to affect older individuals and presents with abrupt beta-cell destruction, often manifesting as DKA with only modest elevation of glycated haemoglobin, reflecting the short interval between beta-cell loss and clinical decompensation [4,5]. The majority of published cases have involved PD-1 inhibitors such as nivolumab and pembrolizumab; however, a growing body of evidence confirms that PD-L1 blockade with atezolizumab carries equivalent risk. Cases arising in the specific context of HCC remain sparsely reported, despite atezolizumab-bevacizumab being the standard first-line regimen for this population, making characterisation of this irAE in the HCC setting of particular clinical relevance [4,5].

We present a case of atezolizumab-associated autoimmune diabetes with DKA in a woman with HCC receiving atezolizumab-bevacizumab combination therapy, accompanied by strongly positive dual islet autoantibodies and early preserved but declining endogenous insulin secretion.

## Case presentation

A 58-year-old woman with hepatocellular carcinoma was referred for palliative immunotherapy. The oncology team commenced atezolizumab and bevacizumab on 3 July 2025, with a scheduled nurse clinic appointment on 16 July 2025. She had no prior personal or family history of diabetes mellitus. Her background included hypothyroidism managed with levothyroxine, with thyroid-stimulating hormone (TSH) within the normal range at the time of presentation.

Osmotic symptoms, progressive polydipsia, polyuria, and general fatigue developed after treatment initiation, with the acute presentation occurring before the planned second cycle of immunotherapy. On assessment, blood tests confirmed diabetic ketoacidosis. There was no precipitating infection, no corticosteroid use, and no history of pancreatitis or alcohol excess. The acute oncology team was notified, with awareness that her next scheduled immunotherapy cycle was due the following week.

Diabetes-specific investigations were performed at the time of the acute presentation (Table 1). Random plasma glucose was 18.4 mmol/L (reference 3.9-7.8 mmol/L). The HbA1c was 53 mmol/mol (reference 20-42 mmol/mol), a finding that, while technically in the diabetic range, was notably modest in the context of frank DKA, consistent with a short window of hyperglycaemia before presentation, a recognised feature of ICI-induced diabetes [4,5]. Islet autoantibody profiling demonstrated strongly positive glutamic acid decarboxylase antibodies (GAD-Ab) at 158 IU/mL (reference <10 IU/mL) and zinc transporter 8 antibodies (ZnT8-Ab) at 1,467 U/mL (reference <15 U/mL). IA-2 antibodies were negative (<10 IU/mL). The urinary C-peptide/creatinine ratio (UCPCR) was 0.59 nmol/mmol (reference 0.2-0.6 nmol/mmol). Interpretation of C-peptide indices during the acute phase of DKA requires caution, as endogenous insulin secretion may be suppressed under conditions of severe metabolic stress; this value is therefore best regarded as a baseline reference point for longitudinal monitoring rather than a reliable indicator of preserved functional beta-cell reserve at the time of acute presentation.

**Table 1:** Biochemical investigations at presentation. GAD, glutamic acid decarboxylase; ZnT8, zinc transporter 8; IA-2, islet antigen-2; TSH, thyroid-stimulating hormone; TPO, thyroid peroxidase

Investigation	Patient result	Reference range
Random plasma glucose	18.4 mmol/L	3.9-7.8 mmol/L
HbA1c	53 mmol/mol	20-42 mmol/mol
Urinary C-peptide	1,760 pmol/L	260-1,730 pmol/L
Urinary creatinine	2.98 mmol/L	2.5-20 mmol/L
Urinary C-peptide/creatinine ratio (UCPCR)	0.59 nmol/mmol	0.2-0.6 nmol/mmol
GAD antibodies	158 IU/mL	<10 IU/mL
ZnT8 antibodies	1,467 U/mL	<15 U/mL
IA-2 antibodies	<10 IU/mL	<10 IU/mL
TSH	0.68 mIU/L	0.4-4.0 mIU/L
TPO antibodies	13.7 kU/L	<35 kU/L
Random serum cortisol	206 nmol/L	172-497 nmol/L

Thyroid function remained stable with a TSH of 0.68 mIU/L (reference 0.4-4.0 mIU/L) and thyroid peroxidase antibodies of 13.7 kU/L (reference <35 kU/L) on background levothyroxine. Random serum cortisol was 206 nmol/L (reference 172-497 nmol/L), within the reference range for a random unstimulated sample. This investigation was performed to exclude a concurrent adrenal immune-related adverse event, which can arise independently in patients receiving ICI therapy. Renal function, liver function tests, and lipid profile were within normal parameters.

The patient was managed according to the local DKA protocol, with intravenous fluid resuscitation, a fixed-rate intravenous insulin infusion, and electrolyte replacement, with resolution of ketoacidosis over the standard treatment window. She was subsequently transitioned to a basal-bolus subcutaneous insulin regimen comprising insulin glargine 16 units once daily and insulin aspart 5 units before each meal. A FreeStyle Libre 3 continuous glucose monitoring sensor (Abbott Diabetes Care Inc., Alameda, CA) was issued before discharge. She received structured diabetes education covering hypoglycaemia recognition and management, sick-day rules with ketone monitoring, and DVLA driving guidance. A follow-up appointment was arranged at the diabetes centre.

Atezolizumab was held during the acute metabolic decompensation, consistent with the approach recommended for grade 3 or higher endocrine toxicity. Oncology was notified and planned to reassess the reinitiation of immunotherapy once glycaemic stability was confirmed on insulin. Given the severity of beta-cell destruction at this stage, lifelong insulin dependence was anticipated regardless of any decision about continuing immunotherapy.

## Discussion

This case represents a well-characterised example of atezolizumab-induced autoimmune diabetes presenting as DKA in the specific oncological context of hepatocellular carcinoma, a combination that remains infrequently reported in the literature. The clinical and immunological profile is instructive on several counts.

The pathophysiological basis for ICI-induced diabetes is thought to involve the physiological role of PD-L1 on pancreatic islet beta cells. Under normal circumstances, PD-L1 expression on islet cells is believed to contribute to the suppression of autoreactive T-cell activity, thereby maintaining a degree of peripheral immune tolerance [2]. Blockade of this interaction by atezolizumab is proposed to lift this inhibitory signal, potentially permitting cytotoxic T-cell infiltration of the islets, a process termed insulitis, with subsequent progressive and often irreversible beta-cell loss [2,4]. It is important to note, however, that the precise causal mechanisms underlying PD-L1 inhibitor-associated diabetes remain incompletely characterised, and multiple parallel immune pathways are likely to be involved. This proposed mechanism shares features with the pathophysiology of spontaneous type 1 diabetes mellitus, though the clinical tempo in ICI-driven cases is typically more rapid.

Two cases of fulminant type 1 diabetes arising specifically during atezolizumab-bevacizumab therapy for HCC have been reported in the Japanese literature. Ikeda et al. described a 71-year-old woman who developed fulminant T1DM with DKA 55 weeks after starting atezolizumab, with blood glucose of 633 mg/dL, undetectable C-peptide, and negative GAD and IA-2 antibodies [4]. The absence of autoantibodies in that case is a recognised feature of fulminant ICI-induced diabetes, where beta-cell destruction is rapid and predominantly T-cell mediated. It should be emphasised that islet autoantibodies, whether GAD-Ab, ZnT8-Ab, or IA-2-Ab, serve as serological markers of autoimmune activation rather than as direct effectors of destruction; cytotoxic T-lymphocyte-mediated insulitis is the principal mechanism of beta-cell loss in both sporadic and ICI-induced autoimmune diabetes. In contrast to the index case reported by Ikeda et al., our patient demonstrated strongly positive dual autoantibodies, confirming the autoimmune aetiology and suggesting a spectrum of immunological mechanisms across ICI-induced diabetes presentations [4,5]. Singhal et al. reported another atezolizumab-treated HCC patient who developed DKA and concurrent Takotsubo cardiomyopathy, a dramatic illustration of the multisystem potential of autoimmune destabilisation by ICI therapy [5].

In the broader atezolizumab literature, Rahman et al. described GAD-antibody-positive type 1 diabetes developing in a patient with presumed pre-existing type 2 diabetes who was receiving atezolizumab for metastatic renal cell carcinoma, highlighting that ICI-induced diabetes can unmask or dramatically accelerate pre-existing latent autoimmune processes [2]. Narumoto et al. reported atezolizumab-induced DKA in a patient with small-cell lung cancer and pre-existing type 2 diabetes, further illustrating that glycaemic decompensation on ICI therapy should not automatically be attributed to worsening of pre-existing diabetes when the presentation is abrupt, and DKA ensues [3].

The disproportionately modest HbA1c (53 mmol/mol) in our patient, despite overt DKA, is a diagnostically important clue that warrants emphasis. It reflects the characteristically short window between beta-cell destruction and clinical presentation in ICI-induced diabetes, a feature that distinguishes it from the gradual metabolic deterioration seen in conventional type 2 diabetes. Clinicians unfamiliar with this pattern may be falsely reassured by a near-normal HbA1c and delay recognition of absolute insulin deficiency. This phenomenon has been consistently observed across reported cases and should reinforce the practice of checking blood ketones alongside plasma glucose whenever osmotic symptoms arise in patients on ICI therapy [4,5].

The UCPCR of 0.59 nmol/mmol, while at the upper limit of the reference range, requires contextual interpretation. As noted above, C-peptide measurement during the acute phase of DKA is subject to the confounding effects of metabolic stress, and values obtained at this point should be interpreted with caution. The UCPCR is a practical and validated non-invasive tool for assessing endogenous insulin secretion, particularly in the outpatient and ward setting where formal stimulated C-peptide testing is not immediately feasible, and serial measurement over subsequent weeks is likely to demonstrate progressive decline as surviving beta cells are eliminated.

From a practical management standpoint, this case underscores several key principles. First, baseline glucose measurement before initiating ICI therapy and pre-cycle glucose checks throughout treatment represent a minimum standard of monitoring; some centres add HbA1c at baseline to facilitate comparison if hyperglycaemia develops. Second, osmotic symptoms, thirst, polyuria, nocturia, and unexplained fatigue in any patient on an ICI should prompt same-day glucose and urine ketone assessment, irrespective of prior diabetes history. Third, the management of ICI-induced diabetes is lifelong insulin therapy; unlike some other irAEs, immunosuppression with corticosteroids does not reverse the pancreatic injury once significant beta-cell loss has occurred [4]. Fourth, the decision to continue, withhold, or permanently discontinue immunotherapy in the context of ICI-DM requires multidisciplinary discussion between oncology and endocrinology, weighing the oncological benefit of continued therapy against the irreversible nature of the endocrine toxicity and the patient's ability to safely manage insulin therapy.

Notably, a parallel pattern has been observed with other PD-L1-based combinations in HCC. Usui et al. recently described fulminant type 1 diabetes developing just three weeks after initiating durvalumab plus tremelimumab for unresectable HCC, a regimen approved for HCC following the HIMALAYA trial and now used alongside atezolizumab-bevacizumab as a first-line option [6]. The rapidity of onset in that case, even shorter than in our patient, reinforces the need for early and regular glucose surveillance from the very first cycle of therapy, regardless of the specific ICI combination being used.

## Conclusions

Atezolizumab-associated autoimmune diabetes is a rare but potentially life-threatening irAE that can arise in the context of HCC treatment with atezolizumab-bevacizumab. The presentation is typically acute, with DKA and near-normal HbA1c, a diagnostic pattern that clinicians should be equipped to recognise. Strongly positive islet autoantibodies, when present, support the immune-mediated aetiology, though their absence does not exclude the diagnosis. Lifelong insulin therapy is required, and the decision regarding continuation of immunotherapy should be made in a collaborative oncology-endocrinology framework. Routine glucose monitoring before and between immunotherapy cycles is essential for early detection and prevention of severe metabolic decompensation.

## References

[1] Finn RS, Qin S, Ikeda M (2020). Atezolizumab plus bevacizumab in unresectable hepatocellular carcinoma. N Engl J Med.

[2] Rahman W, Conley A, Silver KD (2020). Atezolizumab-induced type 1 diabetes mellitus in a patient with metastatic renal cell carcinoma. BMJ Case Rep.

[3] Narumoto K, Oda N, Mitani R, Takata I (2024). Atezolizumab-induced type 1 diabetic ketoacidosis in a patient with small cell lung cancer and pre-existing type 2 diabetes mellitus. Cureus.

[4] Ikeda M, Tamada T, Takebayashi R (2023). Development of fulminant type 1 diabetes mellitus in the course of treatment with atezolizumab for hepatocellular carcinoma. Intern Med.

[5] Singhal S, Patel G, Singh RB, Goyal A, Avgush K, Koka J (2022). Atezolizumab-induced autoimmune diabetes mellitus presenting as diabetic ketoacidosis and Takotsubo cardiomyopathy. BMJ Case Rep.

[6] Usui N, Imai Y, Sugawara K (2025). Hepatocellular carcinoma complicated by fulminant type 1 diabetes mellitus shortly after initiation of durvalumab plus tremelimumab. Intern Med.

